# Association between dietary inflammation index and frailty among community medical staff

**DOI:** 10.3389/fnut.2026.1661832

**Published:** 2026-02-11

**Authors:** Qinglian Lu, Ming Zong, Zhuo-Jun Xu, Shanshan Yu, Lang Liu, Yang Li, Yating Wu, Chunyan Zhao, Hongxia Li

**Affiliations:** 1 Shanghai East Hospital, School of Medicine, Tongji University, Shanghai, China; 2 Department of Clinical Laboratory, Shanghai East Hospital, Tongji University, Shanghai, China; 3 Neuroscience ICU (Stroke ICU), Shanghai East Hospital, Tongji University, Shanghai, China; 4 Department of Nursing, Shanghai East Hospital, Tongji University, Shanghai, China; 5 Department of Nursing, Ruijin Hospital Affiliated to Shanghai Jiao Tong University School of Medicine, Shanghai, China; 6 Union Office, Shanghai East Hospital, Tongji University, Shanghai, China

**Keywords:** cross-sectional study, dietary inflammatory index, frailty, healthcare staff, restricted cubic spline

## Abstract

**Objective:**

To investigate the relationship between the Dietary Inflammation Index (DII) and frailty among community medical staff.

**Methods:**

A stratified cluster sampling method was used to select community healthcare staff from Pudong New Area, Shanghai, between March and September 2024. Demographic characteristics were collected using a general information questionnaire. Dietary nutrient intakes were obtained via 24-hour dietary recall, and DII was calculated. Frailty status was assessed using the Fried Frailty Phenotype Scale. Logistic regression was performed to analyze the correlation between DII and frailty, and a restricted cubic spline model was used to explore the dose–response relationship.

**Results:**

Among 377 healthcare staff, 23 (6.1%) were frail, and 227 (60.2%) were pre-frail. The DII scores ranged from −4.02 to 3.73. The frail group had significantly higher DII scores than the pre-frail and non-frail groups, with statistical significance in DII quartile grouping. Logistic regression analysis showed that for each unit increase in DII absolute value, the risk of frailty increased approximately 3.4 times after adjusting for covariates (95% CI: 1.439 ~ 8.198, *p* = 0.005). The risk of frailty in the high DII group was 1.9 times that of the low DII group (95% CI: 1.152 ~ 3.190, *p* = 0.012). The restricted cubic spline model indicated a non-linear relationship between DII and frailty (*p* = 0.001).

**Conclusion:**

Frailty among community healthcare medical staff is influenced by DII. Maintaining DII within a reasonable range may help prevent frailty.

## 1 Introduction

With the deepening reform of the primary medical and health service system, community medical staff, as the core force of primary medical care, assume multiple responsibilities such as disease prevention, diagnosis and treatment, and chronic disease management (1). However, long-term high-intensity workload, complex doctor-patient relationship and limited development space make them physically and mentally exhausted (2). A global systematic review on occupational burnout among medical staff shows that the burnout rate among medical workers is as high as 44%, making them a key population of concern regarding occupational burnout (3). Studies have confirmed that continuous stress not only affects personal health, but also causes a decline in immune function, a decrease in physical function, increases the risk of physical and mental diseases (4), and makes the body enter a state of frailty in advance (5). Frailty refers to a clinical syndrome in which individuals have reduced physiological reserves and multi-system dysfunction, leading to weakened stress resistance and increased vulnerability (6). At present, the average prevalence of frailty in adults is between 8.2 and 13% (7, 8), and it is closely related to various adverse health outcomes such as chronic fatigue, decreased muscle mass, and occupational burnout (9, 10). Early identification of modifiable factors is crucial for preventing or reversing the occurrence of frailty.

In recent years, studies have found that chronic low-grade inflammation is a key driving factor for the occurrence of frailty (11). Diet, as an important potential source of pro-inflammatory and anti-inflammatory compounds, is an important regulator of inflammation (12). The Dietary Inflammation Index (DII), as a tool to evaluate the overall dietary inflammatory effect, has been used to explore the internal relationship between diet and frailty (13). A higher DII value indicates a greater risk of frailty, suggesting the important role of inflammation in the occurrence of frailty (14). Studies have shown that pro-inflammatory diets can activate the NF-κB pathway and NLRP3 inflammasomes, promote the release of inflammatory factors such as TNF-*α* and IL-6, and accelerate muscle loss and metabolic disorders through mechanisms including chronic low-grade inflammation, oxidative stress, and insulin resistance, thereby increasing the risk of frailty (15). In contrast, anti-inflammatory diets directly scavenge free radicals, inhibit the NF-κB and MAPK signaling pathways, regulate the intestinal microbiota, reduce the entry of lipopolysaccharides (LPS) into the bloodstream, lower systemic inflammation levels, and delay the progression of frailty (16). However, existing studies mostly focus on surveys of the elderly or patients with diseases. As health guardians, the frailty status of medical staff is often easily ignored. Therefore, this study explores the relationship between DII and frailty in medical staff, which can not only identify and prevent the occurrence of frailty early, but also provide a theoretical basis for formulating scientific dietary intervention strategies, which is helpful to maintain the health of medical staff and improve their work ability and quality of life.

## 2 Materials and methods

### 2.1 Patient population

Using the stratified cluster sampling method, some medical staff in a certain area were selected as the research objects from March to September 2024. According to the local geographical location, it was divided into 3 layers: urban area, urban–rural junction and suburban area. The sample size formula was used to obtain the number of people needed for each layer (17). The confidence level was set at 95%, u = 1.96, and the allowable error was 0.05. Based on the incidence of middle-aged frailty in the HANDLS study, P was determined to be 7.2% (18). The sample size of each layer was calculated to be 103 cases. Considering 10% of invalid questionnaires, the sample size of each layer was approximately 113 cases, and finally the sample size of the three layers was 339 cases. The participants who met the inclusion criteria in each layer were selected by the random number table method. In this study, 9 community hospitals were finally selected, including 4 in urban areas: A Community Hospital, B Community Hospital, C Community Hospital and D Community Hospital; 2 in urban–rural junction: E Community Hospital and F Community Hospital; and 3 in suburban areas: G Community Hospital, H Community Hospital and I Community Hospital. Inclusion criteria: doctors, nurses, medical technicians and administrative staff aged ≥ 40 years; working years ≥ 2 years; agreeing to participate in this study. Exclusion criteria: those who are on leave due to illness/injury, maternity leave, assigned to further study or training and other non-on-the-job personnel. A total of 400 questionnaires were distributed in this study, and 377 valid questionnaires were recovered, with an effective recovery rate of 94.25%. This study has been approved by the Ethics Committee (NO: 2024 YS-199), and all participants signed the informed consent form.

### 2.2 General information questionnaire

Self-designed by the researchers, it mainly includes: gender, age, educational, smoking, drinking, daily working hours, whether they are in a state of high pressure or anxiety for a long time, whether they often take physical exercise, average daily sleep time, whether they often stay up late, history of hypertension, history of hyperlipidemia, history of diabetes, and history of cardiovascular and cerebrovascular diseases of medical staff.

### 2.3 Food frequency questionnaire (FFQ25)

FFQ25 was simplified by Chinese nutritionists such as Gao Jian based on previous foreign dietary questionnaires (19). It includes 25 food items/categories, specifically: rice, porridge, flour-based foods, sweets, fried foods, stuffed foods, whole grains, potatoes, dairy products, eggs, red meat, poultry, processed meat products, freshwater fish, seafood, soy products, nuts, dark-colored vegetables, light-colored vegetables, mushrooms, fruits, sweet beverages, beer, yellow rice wine, and white liquor. The frequency of food intake was categorized into the following ranges: never consumed, <1 time/month, 1–3 times/month, 1–2 times/week, 3–4 times/week, 5–6 times/week, 1 time/day, 2 times/day, and ≥3 times/day. The amount of food consumed per time was generally divided into 6 levels, measured in *liang* (a traditional Chinese unit of weight, 1 *liang* ≈ 50 grams): ≤1 *liang*, 2 *liang*, 3 *liang*, 4 *liang*, ≥5 *liang*, and not applicable.

The calculation results yield the average daily intake of each food category over the past month. This scale has been validated among middle-aged and elderly residents in Shanghai, demonstrating good reliability and validity (20).

### 2.4 DII calculation

The DII calculation method designed by Shivappa et al. (13) was adopted, which evaluated the inflammatory effects of 45 nutrients and was usually used to assess dietary inflammation. In this study, 25 nutritional parameters were selected, including energy, protein, fat, carbohydrate, dietary fiber, cholesterol, vitamin A, *β*-carotene, thiamine, riboflavin, niacin, vitamin C, vitamin E, magnesium, iron, zinc, selenium, folic acid, saturated fatty acid, monounsaturated fatty acid, polyunsaturated fatty acid, vitamin B6, vitamin B12, omega-6 fatty acid and omega-3 fatty acid. The specific calculation is as follows: (1) Calculate the Z-score of each of the 25 parameters by subtracting the global standard mean from the representative global diet database from the actual intake, and then dividing by the global standard deviation. (2) Convert the estimated Z-scores to percentiles to minimize the impact of skewness or outliers. These percentiles are centered at 0 (resulting in a symmetric distribution) by multiplying each percentile value by 2–1. (3) The parameter-specific DII score is determined by multiplying the centered percentile value by the corresponding overall food parameter-specific inflammatory effect score, and the overall DII score is obtained by summing all parameter-specific DII scores. A higher DII score indicates a more pro-inflammatory diet, while a lower score indicates a more anti-inflammatory diet.

### 2.5 Frailty assessment

The Fried Frailty Scale developed by Fried et al. (21) was used, which included five aspects: weight loss, self -perceived fatigue, decreased physical activity, slowed walking speed and decreased grip strength. The total score ranges from 0 to 5. A score of < 1 indicates no frailty, 1 ~ 2 points indicate pre-frailty, and ≥ 3 points indicate frailty.

### 2.6 Data collection

Before data collection, investigators were trained uniformly. After obtaining the consent of the hospital director, the investigators explained the purpose, significance and precautions of this study to the participants, and distributed the informed consent form after obtaining their consent. Three investigators worked in coordination: coordinating participants, organizing training and distributing gifts, following up the progress of questionnaires, collecting indicators such as grip strength and walking speed with professional equipment, and rechecking the questionable contents of the questionnaires and measured data on site to ensure the completeness and accuracy of the data.

### 2.7 Statistical analyses

SPSS26.0 and R4.1.2 software were used for statistical analysis. Measurement data conforming to normal distribution were described by mean ± standard deviation, measurement data with skewed distribution were described by median and quartile, and count data were described by frequency and composition ratio. One-way ANOVA was used for comparison between groups. Logistic regression model was used to analyze the relationship between frailty and DII, DII quartiles. Restricted cubic spline (RCS) was used to evaluate the nonlinear relationship between DII and frailty.

## 3 Results

### 3.1 Characteristics of participants

A total of 377 medical staff were included in this study, among whom 64 were male and 313 were female. Among the 377 medical staff, 23 cases of frailty were detected, with the incidence of frailty among medical staff being 6.1%. They were divided into the frailty group (*n* = 23), pre-frailty group (*n* = 227) and non-frailty group (*n* = 127) according to whether frailty occurred. There were statistically significant differences among the three groups of medical staff in terms of educational, whether they took physical exercise, history of diabetes, and history of cardiovascular diseases (*p* < 0.05) (see Table 1).

**Table 1:** Univariate analysis of frailty in medical workers.

Item	Name	Frailty classification [*n* (%)]			*χ* ^2^	*p*
		No frailty	Pre-frailty	Frailty		
Gender	Male	24 (18.898)	34 (14.978)	6 (26.087)	2.33	0.312
	Female	103 (81.102)	193 (85.022)	17 (73.913)		
Age (years)	41–45	49 (38.583)	88 (38.767)	10 (43.478)	3.245	0.778
	46–50	36 (28.346)	70 (30.837)	6 (26.087)		
	51–55	27 (21.260)	54 (23.789)	5 (21.739)		
	56–60	15 (11.811)	15 (6.608)	2 (8.696)		
Educational	Below junior college	29 (22.835)	47 (20.705)	3 (13.043)	16.502	0.011*
	Junior college	15 (11.811)	61 (26.872)	2 (8.696)		
	Bachelor	75 (59.055)	105 (46.256)	15 (65.217)		
	Postgraduate and above	8 (6.299)	14 (6.167)	3 (13.043)		
Daily working hours(hours)	<8	100 (78.740)	177 (77.974)	16 (69.565)	2.923	0.818
	8–10	20 (15.748)	42 (18.502)	6 (26.087)		
	10–12	4 (3.150)	5 (2.203)	1 (4.348)		
	>12	3 (2.362)	3 (1.322)	0 (0.000)		
High pressure or anxiety	No	100 (78.740)	167 (73.568)	15 (65.217)	2.349	0.309
	Yes	27 (21.260)	60 (26.432)	8 (34.783)		
Smoking history	No	115 (90.551)	210 (92.511)	19 (82.609)	2.68	0.262
	Yes	12 (9.449)	17 (7.489)	4 (17.391)		
Drinking history	No	114 (89.764)	213 (93.833)	20 (86.957)	2.706	0.258
	Yes	13 (10.236)	14 (6.167)	3 (13.043)		
Physical exercise	No	80 (62.992)	176 (77.533)	16 (69.565)	8.65	0.013*
	Yes	47 (37.008)	51 (22.467)	7 (30.435)		
Average daily sleep time	<5 h	9 (7.087)	16 (7.048)	1 (4.348)	2.372	0.668
	5-8 h	99 (77.953)	188 (82.819)	20 (86.957)		
	>8 h	19 (14.961)	23 (10.132)	2 (8.696)		
Whether often stay up late	No	44 (34.646)	100 (44.053)	9 (39.130)	3.01	0.222
	Yes	83 (65.354)	127 (55.947)	14 (60.870)		
History of hypertension	No	101 (79.528)	185 (81.498)	15 (65.217)	3.451	0.178
	Yes	26 (20.472)	42 (18.502)	8 (34.783)		
History of hyperlipidemia	No	92 (72.441)	149 (65.639)	15 (65.217)	1.81	0.405
	Yes	35 (27.559)	78 (34.361)	8 (34.783)		
History of diabetes	No	122 (96.063)	220 (96.916)	19 (82.609)	10.564	0.005**
	Yes	5 (3.937)	7 (3.084)	4 (17.391)		
History of cardiovascular and cerebrovascular diseases	No	121 (95.276)	207 (91.189)	18 (78.261)	7.731	0.021*
	Yes	6 (4.724)	20 (8.811)	5 (21.739)		

*p < 0.05; **p < 0.01.

### 3.2 DII score status

The DII score of 377 medical staff was 2.36 (−4.02 ~ 3.73) points. The DII score in the frailty group was higher than that in the pre-frailty group and the non-frailty group, with a statistically significant difference (*χ*^2^ = 11.456, *p* < 0.001). There was also a statistically significant difference among the three groups of medical staff in terms of DII quartile grouping (*χ*^2^ = 18.469, *p* = 0.005) (see Table 2).

**Table 2:** Comparison of DII among medical staff.

Item	No Frailty (*n* = 127)	Pre-frailty (*n* = 227)	Frailty (*n* = 23)	Statistical Value	*p*
DII	1.804 ± 0.883	2.203 ± 0.801	2.396 ± 0.635	11.456	0.000**
Grouping (*n*, %)					
Q1	42 (33.071)	48 (21.145)	4 (17.391)	18.469	0.005**
Q2	37 (29.134)	54 (23.789)	4 (17.391)		
Q3	32 (25.197)	56 (24.670)	7 (30.435)		
Q4	16 (12.598)	69 (30.396)	8 (34.783)		

**p < 0.01.

### 3.3 Logistic regression analysis of DII and frailty

Taking whether medical staff suffered from frailty as the dependent variable, and DII score and DII quartile as independent variables respectively, logistic regression analysis was conducted with variables with statistical significance in univariate analysis as covariates. The results showed that there was a significant positive correlation between DII and the risk of frailty. Specifically, for each increase in the absolute value of DII by one unit, the risk of frailty increased by approximately 3.4 times after adjusting for covariates (OR = 3.435, 95%CI: 1.439 ~ 8.198, *p* = 0.005); when analyzed by DII quartile, compared with the lowest inflammatory diet group (Q1), for each increase in DII by one unit, the risk of frailty still significantly increased by approximately 1.9 times after adjusting for covariates (OR = 1.917, 95%CI: 1.152 ~ 3.190, *p* = 0.011) (see Table 3).

**Table 3:** Relationship between DII and frailty [OR (95% CI)].

Variable	OR	*p*	OR	*p*
Absolute value of DII	Model 1		Model 2	
	2.916 (1.373 ~ 6.192)	0.005	3.435 (1.439 ~ 8.198)	0.005
DII quartile	Model 3		Model 4	
	1.799 (1.159 ~ 2.793)	0.009	1.917(1.152 ~ 3.190)	0.012

Covariates were not added in Model 1 and Model 3; Covariates were added in Model 2 and Model 4.

### 3.4 Analysis of nonlinear relationship between DII and frailty

**Figure 1:**
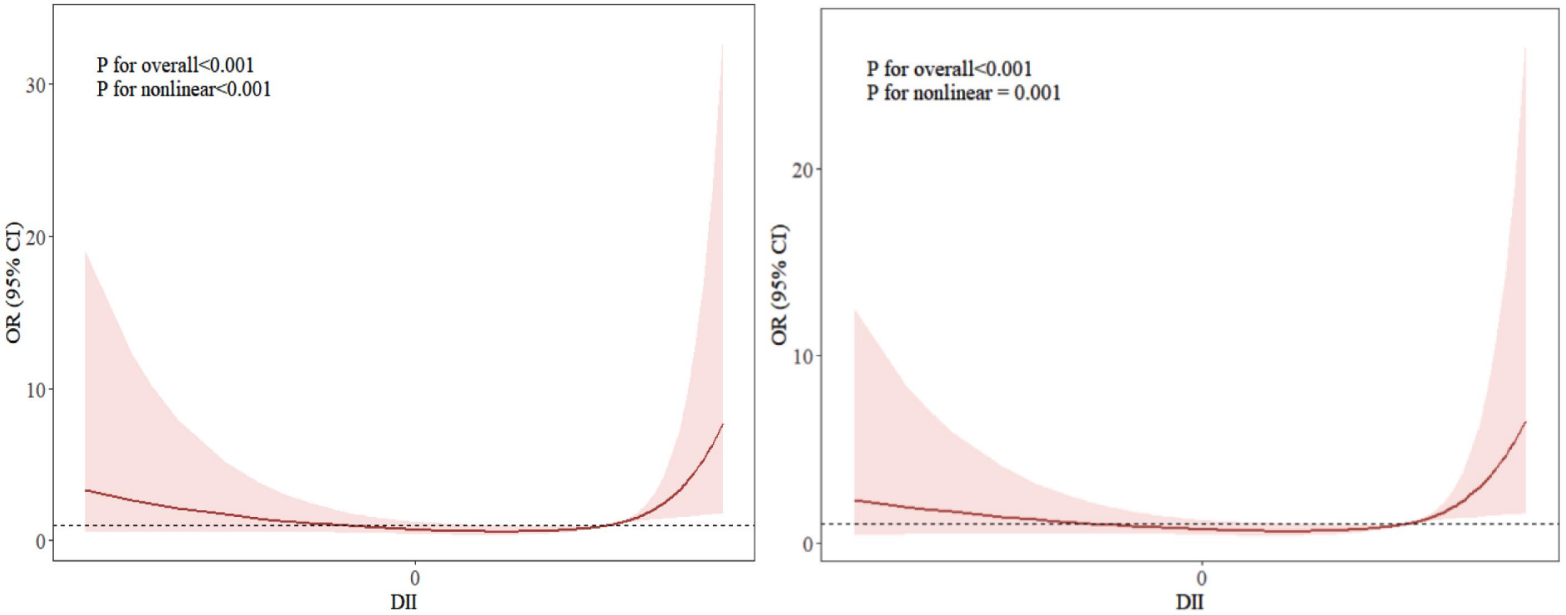
Dose–response relationship curve between DII and frailty. The right side without any covariates added; the left side with covariates adjusted, including educational background, whether to take physical exercise, history of diabetes, and history of cardiovascular diseases.

Taking the frailty score as the dependent variable and DII as the independent variable, a restricted cubic spline model was established after adjusting for covariates such as educational, whether to take physical exercise, history of diabetes, and history of cardiovascular diseases. The results showed a nonlinear relationship between DII and frailty (nonlinear *p* = 0.05). The risk of frailty in medical workers showed a “U-shaped” trend with changes in DII. Specifically, when DII was in the two intervals of < −0.95 and > 2.36, the risk of frailty increased significantly; while when DII was in the interval of −0.95 ~ 2.36, there was a negative correlation between DII and the risk of frailty (see Figure 1).

## 4 Discussion

### 4.1 Community medical workers are at risk of frailty, with most in the pre-frailty stage

The results of this study showed that the incidence of frailty among community medical workers was 6.1%, and the incidence of pre-frailty was as high as 60.2%, which was higher than the survey on frailty (3.1%) and pre-frailty (40%) among ordinary Chinese adults by Fan et al. (22). This may be closely related to the unique occupational stress, working environment and lifestyle of medical workers. The state of frailty and pre-frailty not only directly damage the physical and mental health of medical workers, increase their risk of suffering from other diseases, but also may indirectly lead to the impairment of their health productivity (23) and reduce the overall quality of primary medical services. In view of the negative impact of frailty on medical staff, medical institutions and relevant departments should attach great importance to the health status of medical staff, take targeted intervention measures to help medical staff in the pre -frailty stage reverse to a healthy state, so as to reduce or delay the risk of frailty and ensure the health and stability of the primary medical team.

### 4.2 The overall DII level of community medical workers is relatively high

The results of this study showed that the DII score of community medical workers was 2.36 (−4.02 ~ 3.73). As a widely used tool for evaluating dietary inflammation, DII can objectively quantify the overall pro-inflammatory or anti-inflammatory potential of an individual’s dietary pattern (24). In this survey, the overall DII of medical workers was at a relatively high level, suggesting that the daily dietary structure of this group may tend to include a higher proportion of pro-inflammatory foods, such as refined grains, high-sugar foods, red meat and processed meat, and foods rich in saturated fat and trans fat, while the intake of anti-inflammatory foods, such as dark leafy vegetables, fruits, whole grains, fish rich in Omega-3 fatty acids and nuts, was relatively insufficient (25). This may be because medical staff often face factors such as high-intensity work pressure, irregular work and rest (such as shift work and night shifts), and limited dining conditions (urgent meal time, reliance on fast food or takeout, limited choices in hospital canteens) (26), which may prompt them to choose convenient but low-nutritional value and high pro-inflammatory potential foods. Maintaining this dietary pattern for a long time may have an adverse impact on the body’s chronic inflammatory state. Therefore, it is suggested that medical staff should actively adjust their dietary structure, consciously increase the proportion of anti-inflammatory foods and reduce the intake of pro-inflammatory foods to help reduce the level of chronic inflammation in the body. This not only helps them prevent or delay frailty, maintain long-term health and work ability, but also helps them guide patients to develop healthy eating habits, arrange their dietary structure reasonably, and reduce the risk of frailty.

### 4.3 DII is correlated with the risk of frailty and there is a nonlinear relationship

The results of Logistic regression analysis showed that the higher the DII score, the greater the risk of frailty, which was similar to the research results of Li et al. (27). After adjusting for 4 covariates, the incidence of frailty in the high DII group was significantly 1.9 times higher than that in the low DII group, indicating that DII is a risk factor for frailty in medical workers. Zhao et al. (28) also confirmed in patients with osteoarthritis that each increase in DII by one standard deviation was associated with a 15% increase in the risk of frailty, and that patients with DII > 1 had a significantly higher risk of frailty compared with those with DII < −1. Studies have found that long-term excessive intake of pro-inflammatory dietary components such as refined carbohydrates, fried foods, excessive *ω*-6 fatty acids, high-sugar drinks and Baijiu can induce an increase in the level of inflammatory biomarkers in the body (29). These inflammatory factors can not only promote muscle protein decomposition and accelerate the decline of body tissue function, but also significantly increase the risk of frailty by activating systemic inflammatory response and interfering with neuroendocrine homeostasis (30, 31).

Further analysis through restricted cubic spline in this study revealed a significant nonlinear association between DII and the risk of frailty (*p* < 0.001), which was consistent with the research results of Lin et al. (32). The dose–response curve indicated that beyond a certain threshold, both higher anti-inflammatory and pro-inflammatory levels were associated with an increased risk of frailty. The potential reasons are as follows: A highly pro-inflammatory diet may induce gut microbiota dysbiosis (33), which not only directly impairs intestinal barrier integrity and mucosal immune homeostasis, leading to endotoxemia, but also regulates central inflammatory responses via the gut-brain axis and disseminates inflammatory signals through the bloodstream. This creates a vicious cycle of “gut microbiota dysbiosis-systemic inflammation-tissue functional decline,” further accelerating the progression of frailty (34). Conversely, an extremely anti-inflammatory diet may suppress normal immune responses, disrupt the balance of gut microbiota diversity, or interfere with the balanced supply of key nutrients such as fatty acids and amino acids. This could lead to reduced metabolic adaptability, impaired tissue repair, and ultimately chronic low-grade inflammation or metabolic disorders, thereby increasing frailty risk (35, 36). Maintaining DII within an appropriate range (−0.95 to 2.36) through dietary patterns may help sustain a dynamic balance between inflammatory responses and gut microecology, avoiding the disruption of metabolic homeostasis caused by excessive pro-inflammatory or anti-inflammatory effects. This could serve as a key intervention strategy to reduce frailty risk. This implies that appropriate dietary adjustments within this range, while avoiding prolonged exposure to highly pro-inflammatory or anti-inflammatory states beyond the threshold, are more conducive to lowering the risk of frailty.

This study has the following limitations. Firstly, this study adopted a cross-sectional design, which cannot infer the causal relationship between the DII and frailty. Future studies should employ longitudinal designs such as prospective cohort studies to clarify the long-term predictive effect of DII on frailty risk. Secondly, this study had limited control over confounding factors. Potential confounding variables such as health status, psychological factors (e.g., occupational burnout, work stress), sleep-related indicators (e.g., sleep quality, sleep duration), and individual dietary preferences were not fully incorporated into the analysis. Coupled with the constraints of a single-region sample, the external validity of the findings was compromised. For future research, it is recommended to systematically identify potential confounding factors associated with dietary inflammation and frailty based on relevant theoretical frameworks, incorporate a more comprehensive set of variables into the analytical model, and conduct multi-center analyses and validation to address these limitations. Thirdly, the dietary intake data in this study relied on retrospective surveys, which may introduce recall bias and affect the accuracy of dietary intake data. To address this, future studies can add graphical reference tools for food portions in questionnaires, assist participants in clearly recalling dietary details through standardized guided interviews, and reduce issues such as omissions of high-frequency foods and errors in intake estimation. Meanwhile, it is recommended to combine more comprehensive dietary assessment methods such as 3-day consecutive dietary records and Food Frequency Questionnaires (FFQ) to further improve the accuracy of DII calculation.

## 5 Conclusion

The results of this study showed that the incidence of frailty among medical workers was relatively low, and most of them were in the pre-frailty stage. There is a non-linear dose–response relationship between DII and frailty. When DII is maintained within a reasonable range, the risk of frailty occurrence decreases. The research results supplemented the research content in the field of frailty for the special group of medical workers, and provided a reference basis for preventing frailty and optimizing the dietary management of medical workers. It is suggested that medical workers should maintain a balanced diet, appropriately increase the intake of anti-inflammatory diets, reduce the intake of pro-inflammatory foods, and control DII within an appropriate range.

## Data Availability

The original contributions presented in the study are included in the article/Supplementary material, further inquiries can be directed to the corresponding authors.
